# *FTO* rs9939609 Does Not Interact with Physical Exercise but Influences Basal Insulin Metabolism in Brazilian Overweight and Obese Adolescents

**DOI:** 10.1155/2018/3134026

**Published:** 2018-04-26

**Authors:** Gabrielle Araujo do Nascimento, Neiva Leite, Lupe Furtado-Alle, Mayza Dalcin Teixeira, Ricardo Lehtonen Rodrigues de Souza, Gerusa Eisfeld Milano, Larissa Rosa da Silva, Juliana Pizzi, Wendell Arthur Lopes, Maria de Fátima Aguiar Lopes, Ana Cláudia Kapp Titski, Luciane Viater Tureck

**Affiliations:** ^1^Department of Genetics, Centro Politécnico, Setor de Ciências Biológicas, Federal University of Paraná, Francisco H. dos Santos, 210 Jardim das Américas, 81531-970 Curitiba, PR, Brazil; ^2^Department of Physical Education, Federal University of Paraná, Coração de Maria, 92 Jardim Botânico, 80215-370 Curitiba, PR, Brazil; ^3^Department of Physical Education, State University of Maringa, Av. Colombo 5790, 87020-900 Maringa, PR, Brazil; ^4^Academic Department of Education, Federal University of Technology–Paraná, Av. Monteiro Lobato Km 04, 84016-210 Ponta Grossa, PR, Brazil

## Abstract

**Purpose:**

The rs9939609 SNP (T > A) in *FTO* gene is associated with obesity and type 2 diabetes. The present study aimed at verifying whether this SNP influenced biochemical outcomes of children and adolescents who are overweight/obese submitted to a program of physical exercise and also if there was influence on basal levels of these biochemical variables.

**Methods:**

The sample was composed by 432 children and adolescents grouped in three ways (obese, overweight, and normal weight); of these, 135 children and adoloescents who are obese and overweight were submitted to a physical exercise program for 12 weeks. All were genotyped by TaqMan SNP genotyping assay.

**Results:**

The children and adolescents who are overweight/obese and carriers of AA genotype had higher levels of insulin (*p*=0.03) and HOMA (*p*=0.007) and lower levels of glucose (*p*=0.003), but the SNP did not modulate the response to physical exercise.

**Conclusions:**

In our study, the rs9939609 AA genotype was associated with parameters related to insulin metabolism but did not interact with physical exercise.

## 1. Introduction

Obesity is a multifactorial disease influenced by several genetic factors, including the fat mass and obesity-associated (*FTO*) gene [1, 2], which is expressed in the whole organism, mainly in hypothalamus, and thus associated with energy balance regulation [3]. *FTO* gene product is a 2-oxoglutarate dependent nucleic acid demethylase [4] which can have several target genes, including genes related to metabolism.

One of its most studied SNPs is the rs9939609 SNP (T > A), associated with obesity, type 2 diabetes, and other metabolic complications [1, 5, 6]. The body weight increase associated with rs9939609 A-allele could be related to an increase in *FTO* expression, since *FTO* transcripts are more abundant in risk allele carriers [7].

Many studies have shown association between A-allele and increased food intake and reduced satiety [8–11]; however, the interaction of the rs9939609 SNP with energy expenditure has not yet been clarified, since some studies found interaction and others did not [12].

Besides the effect induced by rs9939609 A-allele in several basal metabolic parameters [13–16], it can also interfere in the response promoted by physical and/or dietary interventions. Some studies have found a reduction in levels of cholesterol, low-density lipoprotein cholesterol (LDL-C), insulin, and HOMA-IR in A-allele carries in response to diet [17, 18]. Regarding the physical exercise, any study that analyzed the interaction of the rs9939609 A-allele on biochemical outcomes was not found.

Efforts have been adopted to understand the interactions between genetic and environmental factors underlying the complex pathologies. In this sense, the identification of modifiable factors that may contribute to decrease the genetic predisposition on metabolic disorders is important for prevention and treatment. Considering the potential influence of rs9939609 on biochemical variables that predict essential metabolic functions, and the lack of studies with *FTO* and physical exercise interaction, the present study investigated the rs9939609 SNP (T > A) effect on biochemical outcomes induced by physical exercise in children and adolescents who are overweight/obese.

## 2. Materials and Methods

### 2.1. Subjects

The study was composed by 432 children and adolescents (290 boys and 142 girls), of which 169 had normal weight and 263 had overweight or were obese (80 overweight and 183 obese), according to parameters defined by WHO [19]. The mean overall age was 13.51 ± 0.09 years old (aged 8–17 y), and the mean BMI *Z*-score in the overweight/obese group was 2.69 before the exercise and 2.73 after the exercise. In the normal weight group, the mean BMI *Z*-score was −0.22. This study was limited to analysis of the biochemical variables in these individuals. Some of them were analyzed in a previous study that was limited to anthropometric variables analysis [20].

The recruitment of children and adolescents was carried out in public schools of the state of Paraná, Southern Brazil, with the following inclusion criteria: children and adolescents who have medical liberation for physical exercise and do not use drugs that could interfere on weight control and/or lipid levels. An invitation to participate in the research was made to those individuals who met the criteria and those who accepted signed the free and informed consent term, along with the legal responsible consent [20]. The study was approved by the ethics committee of the Federal University of Paraná (UFPR) (protocol number 765.184/2003-11) [20].

Weight and height were measured with an accuracy of 0.1 kg and 0.1 cm, respectively. BMI was calculated as weight in kilograms divided by the square of height in meters and then converted into BMI *Z*-score according to WHO [19]. The children and adolescents were considered overweight when their BMI *Z*-score was between +1 and +2 and obese when their BMI *Z*-score was more than +3 [19].

### 2.2. Biochemical Variables

The blood samples were collected, and total cholesterol (TC), HDL-C, and TG were measured by standard procedures in private partner laboratories and in the clinical analyzes laboratory of UFPR. Blood glucose levels were determined by the enzymatic method, and insulin was measured by the chemiluminescence immunoassay technique by automated equipment. LDL-C levels were calculated using the Friedewald equation [21], homeostatic model assessment for insulin resistance (HOMA-IR) was calculated using the formula fasting blood glucose (*µ*U/ml) × insulin (mMol/l)/22.5 [22], and the quantitative insulin sensitivity check index (QUICKI) was calculated using the formula 1/(log (fasting insulin) (mU/ml) × log (fasting blood glucose) (mMol/l)) [23]. These variables were measured before and after the exercise program.

### 2.3. Physical Exercise Intervention Program

Of the 263 children and adolescents who are overweight or obese and who participated in the study, 135 were submitted to an after-school exercise program. This research is part of a larger project the objective of which was to analyze the response of the children and adolescents who are overweight or obese to different physical exercises [24–27].

The exercise intervention programs were carried out in a special session offered in the school gym (land-based aerobic exercise, high-intensity interval training (HIIT), and combined training) or in the university setting (water walking).

Each student participated in one of the four different physical exercise programs: land-based aerobic exercise, HIIT, combined training, or water walking. Each physical exercise program consisted of 3 sessions per week for 12 weeks. They were instructed in the FITT principles (frequency, intensity, time, and type of exercise) by physical education teachers in the school or in the university setting. The minimal adherence accepted was 70% of attendance.

The details of exercise programs are described below and also in previously published works [20,24–27].

The land-based aerobic exercise (*n*=53) was performed in a total of 110 minutes, divided into 45 minutes of walking, 45 minutes of indoor cycling, and 20 minutes of stretching. The reserve heart rate (RHR) was accessed, with 35% to 55% in the first to fourth week, 45% to 65% in the fifth to eight week, and 55% to 75% in the last weeks [24].

HIIT (*n*=27) was performed in 45 minutes in each session, and the students did warming-up exercises, running for 30 seconds at 100% speed peak effort, walking for 60 seconds at 50% peak velocity (active recovery period), and relaxing. The exercise was composed by two sets, and there were four minutes of rest between sets. The training progressed as the weeks passed: in the first week, the set was composed by 4 × 30 s/60 s; in the week 2, it was composed by 5 × 30 s/60 s; in the week 3, it was 6 × 30 s/60 s; in the weeks 4 and 5, it was 7 × 30 s/60 s; in the weeks 6 to 9, the recovery period was reduced to 45 s (8 × 30 s/45 s); and in the last two weeks, it was 8 × 30 s/30 s [25].

The combined training (*n*=29) was a combination of resistance and aerobic training, and each session lasted 60 minutes. In the resistance training, the students realized six exercises: leg press, leg extension, leg curl, bench press, lateral pull down, and arm curl. It was composed by three sets of 6–10 repetitions at 60–70% 1 RM (maximum repetition), and the load was adjusted weekly. In the aerobic training, the students walked/ran in an athletic track for 30 minutes, and the intensity was 50–80% of VO_2peak_ [26].

The water-walking exercise (*n*=26) was realized with the support of a float vest attached to the waist, and it is a simulation of walking on land without the contact of the feet with the bottom of the pool. The students realized five minutes of warming up, 45 minutes of walking, and 10 minutes of recovering, totalizing 60 minutes in a session. The exercise intensity was 40% to 60% of the RHR and increased in the fifth and ninth weeks [27].

In the statistical analyzes, these groups were analyzed together due to the small number of participants in each one; however, in some analyzes, the type of exercise was added as a correction factor. The analysis of some variables was realized with a smaller number of individuals, since it was not possible to obtain data on all variables from all individuals who completed the program (*n*=135).

**Figure 1:**
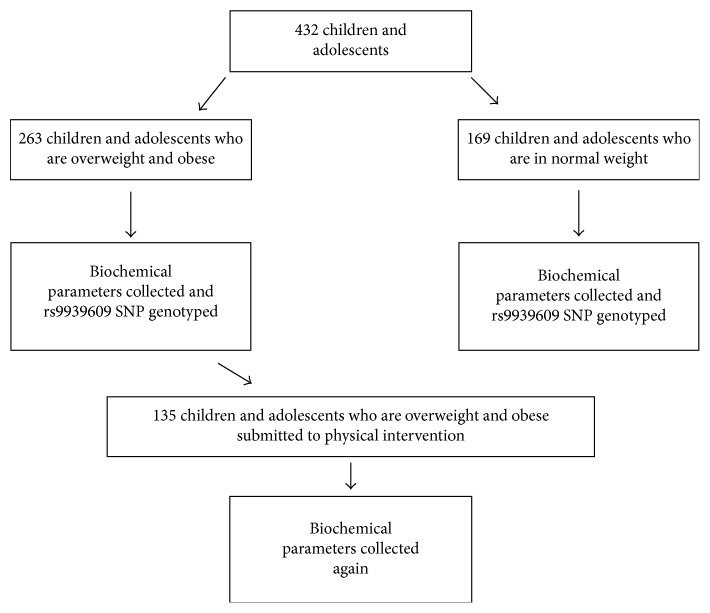
Study design.

The experimental procedure applied is demonstrated in Figure 1.

### 2.4. DNA Extraction and Genotyping

DNA were extracted from peripheral blood according to a salting-out technique [28] and then diluted to 20 ng/*µ*l. *FTO* rs9939609 SNP was genotyped with a TaqMan SNP genotyping assay (Applied Biosystems). The reactions were done using the following conditions: 60°C for 30 s, 95°C for 10 min, 50 cycles of 95°C for 15 s and 60°C for 1 min, and 60°C for 30 s. In each reaction, three previously sequenced control samples were included, representative of each of the possible genotypes.

### 2.5. Statistical Analysis

The frequencies of genotypes and alleles were obtained by direct counting and compared between the group of overweight/obese and normal weight by chi-square test, which was also used to check the Hardy–Weinberg equilibrium. The continuous variables were tested for normality using the Kolmogorov–Smirnov test with the Lilliefors correction. The recessive, dominant, and lack of dominance models of allelic interaction were tested. The recessive model was adopted for analysis that involved the sample stratification by rs9939609 SNP genotype. For each variable, the means were compared between genotypes by parametric or no parametric tests (*t* test or Mann–Whitney, resp.). The comparisons between initial and final means (before and after physical exercise) were conducted by the Wilcoxon test or *t* test for dependent samples.

A two-way mixed ANOVA with repeated measures was used to verify the genotype influence on the response to physical exercise, and the nonparametric variables were normalized by logarithmic transformation. Multiple regression analysis was also applied. Statistical significance adopted for the tests was 0.05 (5%).

## 3. Results

The risk allele (A) was not associated with obesity in our study (overweight/obese and normal weight group with similar allelic frequency *p*=0.71). In the overweight and obese group (*n*=263), 38.02% were TT carriers, 48.67% AT, and 13.31% AA. The genotype frequencies in the normal weight group (*n*=169) were 37.28% (TT), 52.66% (AT), and 10.06% (AA). Both groups are in Hardy–Weinberg equilibrium.

A mixed two-way ANOVA test was performed to investigate possible effects of physical intervention (within subject factor: before and after training); genotype (between subject factor: AA × AT + TT); and training × genotype interaction on the biochemical variables (Table 1). It was possible to observe that the physical intervention and genotype independently influenced the biochemical variables of the children and adolescents who were overweight and obese.

The physical exercise, independently of the *FTO* rs9939609 genotype, improved the metabolic profile: TC, LDL-C, and insulin levels decreased after the intervention (*p*=0.002, *p*=0.02, and *p*=0.02, resp.), and QUICKI increased (*p*=0.03), indicating insulin sensitivity improvement of the children who are overweight and obese (Table 1).

The *FTO* genotype influenced the glucose levels (*p*=0.03) and HOMA-IR index (*p*=0.007) (Table 1). Higher HOMA-IR was associated with AA carriers before and after the physical exercise (*p*=0.006 and *p*=0.01, resp.). The same genotype was associated with lower glucose levels only after the intervention (*p*=0.002) (Supplementary Table 1). According to the ANOVA analysis, the AA carriers trend toward higher levels of insulin (*p*=0.07) after the physical exercise and lower values of QUICKI (*p*=0.07) before and after the physical exercise, compared to TT + AT carriers (Table 1 and Supplementary Table 1), suggesting that AA genotype could be associated with less favorable insulin profiles. There was no genotype × training interaction, since the changes in variables after the intervention were not different among genotypes (Table 1).

To investigate the observed trends in ANOVA and identify other factors that contributed to the insulinic profile of children who are overweight and obese, we conducted multiple regression analysis with the initial measures (before), final measures (after), and also with the differences between final and initial values (difference). All the multiple regression analyses were corrected for age, sex, and BMI *Z*-score. Regression analysis with the final and differences values was corrected for the type of exercise practiced (land-based aerobic exercise, high-intensity interval training (HIIT), combined training, or water walking) (Table 2).

The effect of the *FTO* rs9939609 genotype on the biochemical variables of children and adolescents with normal weight was also evaluated. The comparisons of mean levels of the variables between carriers and noncarriers of AA genotype did not present significant results (Supplementary Table 2), as well as multiple regression analyses (corrected for age, sex, and BMI *Z*-score) (Table 2).

The regression analysis results confirmed the rs9939609 AA genotype effect in children who are overweight and obese, even after the applied corrections. The initial and final insulin levels were influenced by *FTO* SNP (*p*=0.04 and *p*=0.03, resp.), as well as the initial HOMA-IR (*p*=0.01), and final glucose levels (*p*=0.003). The differences in biochemical variables levels (initial levels − final levels) were not influenced by *FTO* SNP, which corroborates the absence of the *FTO* SNP × training interaction, observed in ANOVA.

## 4. Discussion

Our study was designed to investigate the interaction between *FTO* rs9939609 SNP and a program of physical exercise on biochemical outcomes of the children and adolescents who were overweight and obese. In this sense, the interaction between the *FTO* rs9939609 SNP and physical exercise did not affect the biochemical outcomes; however, the *FTO* rs9939609 SNP was associated with insulin, HOMA-IR, and glucose levels of the children and adolescents who were overweight and obese.

Our study identified an important relationship between AA genotype and less favorable insulin profiles: the mean values of insulin in AA individuals were higher than those recommended by some authors [29]. There are at least two possible ways that would explain this relationship, both involving the *FTO* overexpression related to rs9939609 A-allele [7].

The most direct relationship between rs9939609 and insulin resistance is suggested by Tschritter and colleagues [30]. They found association between the *FTO* rs8050136 SNP and cerebrocortical insulin resistance in humans [30], but this polymorphism is in linkage disequilibrium with rs9939609 (rs9939609, *D*′  =  0.9998 [31]); so we cannot rule out a causal effect of the rs9939609 SNP. Tschritter and colleagues observed a reduction of the insulin effect in cortical activity, which decreases the cerebrocortical response to insulin [30]. Therefore, AA genotype carriers may have reduced insulin effect in the brain, which has led to increased insulin production, resulting in higher insulin values and HOMA index compared to AA genotype noncarriers.

After training, the insulin levels remained higher, and the glucose levels in these individuals were significantly lower compared to AA genotype noncarriers. The practice of physical exercise promotes a reduction in glucose levels, since the muscle contractions generate an increase in glucose uptake through an enhancement in glucose transporter type four (GLUT4) production [32, 33]. However, more studies are necessary to understand the interaction between the rs993960AA genotype, exercise, and glucose outcome.

A less direct relationship between rs9939609 and insulin levels may involve the demethylase function of the FTO [4]. Merkestein and colleagues [34] found that male mice with *FTO* overexpression have lower adiponectin levels after 20 weeks, which agrees with the reduction in adiponectin levels found in humans who are obese [35]. Adiponectin is a hormone produced mainly in white adipose tissue and has a negative correlation with obesity. All the adiponectin functions are not known exactly, but it is probably involved in glucose, TG, and fatty acids decrease. Low levels of this hormone increase the susceptibility to insulin resistance and type 2 diabetes [36]. By binding to its receptors (AdipoR1 and AdipoR2), adiponectin activates the AMP-activated protein kinase (AMPK), which stimulates glucose utilization and fatty-acid combustion. Therefore, the reduction in adiponectin levels would impair the glucose metabolism and insulin sensitivity [37, 38]. Thus, the *FTO* rs9939609 SNP can contribute to the adiponectin modulation, and consequently, for the glucose metabolism.

Another possible target of FTO is the *ghrelin* gene. Karra and colleagues [39] found that cells with *FTO* overexpression have increased expression of ghrelin mRNA [39]. Ghrelin is a hormone synthesized by the stomach that promotes increased appetite and food intake and decreased insulin sensitivity, among other functions [40]. Therefore, the effects in glucose metabolism observed in the present study can also be explained by ghrelin overexpression.

Additionally, similar to our findings, other studies also observed association of AA genotype with insulin levels and insulin resistance index [15, 41, 42]. Considering that the *FTO* target genes are not fully known, there is a wide variety of possible genes related to metabolism that may had their expression altered in function of *FTO* rs9939609 genotype.

We did not find any effect of the rs9939609 AA genotype on biochemical outcomes in response to training physical program. The studies regarding interactions between *FTO* and physical exercise have controversial results, since some works found interaction [43, 44] and some did not [3,10,45–47]. Those discrepant results could be attributed to the different samples and different ways to analyze the physical activity. Regarding the samples, some studies included children and adolescents [3, 48] while others involved adults [43–46]. Kilpeläinen and colleagues [49] showed that the age is an important factor that modulates the *FTO* and physical exercise interaction, since they observed the interaction between rs9939609 SNP and physical activity only in adults, and not in children and adolescents [49]. Probably other factors also contribute to the heterogeneity in study results, such as gender, ethnicity, and metabolic status.

Moreover, the method of measuring physical activity can also contribute to the heterogeneity, which requires caution in comparisons. Some authors used questionnaires [44, 45, 48], which also differ between them; and other studies analyzed the maximal oxygen uptake (VO_2_max), resting energy expenditure, and basal metabolic rate [3, 10, 41]. Our study is one of the few that evaluated the rs9939609 effect on biochemical outcomes of the children and adolescents who were overweight and obese submitted to a physical intervention program, which promoted benefits, improving homeostasis regarding CT, LDL-C, insulin, and QUICKI levels.

### 4.1. Limitations

Our work has some limitations, as the sample size, which did not make possible to separate children and adolescents by gender, age, or type of physical exercise. However, the type of physical exercise was included in the multiple regression analysis, adjusting our results to this variable.

### 4.2. Conclusions

In conclusion, we found that the rs9939609 AA genotype influenced parameters related to insulin metabolism and did not interact with physical exercise. Studies that seek to identify the effect of the genetic polymorphisms in metabolic markers are important because they can help with prevention alternatives and more individualized treatments, according to the genetic background of each patient. Additionally, these studies may identify different metabolic pathways in which of these genes participate, and, possibly, indicate new insights into pharmaceutical researches.

## Figures and Tables

**Table 1:** Initial and final averages of biochemical variables stratified by rs9939609 *FTO* SNP genotype and effect of exercise (*p* value training), genotype (*p* value genotype), and training × genotype interaction (*p* value T × G).

Variables	TT + AT (mean ± SD)				AA (mean ± SD)				*p* value		
	*n*	Before	After	%Δ	*n*	Before	After	%Δ	Training	Genotype	T × G
TC (mg/dl)	118	163 ± 35.81	157 ± 32.79	−3.68	17	161 ± 31.75	148 ± 29.26	−8.07	**0.002**	0.55	0.20
HDL-C (mg/dl)	117	48 ± 10.9	46 ± 11.81	−4.17	17	52 ± 11.95	46 ± 9.91	−11.54	**0.005**	0.43	0.05
LDL-C (mg/dl)	118	94 ± 29.5	90 ± 27.42	−4.26	17	92 ± 24.27	83 ± 30.94	−9.78	**0.02**	0.44	0.34
TG (mg/dl)	117	104.44 ± 53.98	102.71 ± 52.84	−1.66	17	85.85 ± 30.35	98.06 ± 68.54	14.22	0.4	0.34	0.26
Glucose (mg/dl)	117	87 ± 9.39	86 ± 8.06	−1.15	18	82 ± 9.6	80 ± 5.76	−2.44	0.07	**0.003**	0.77
Insulin (uIU/ml)	94	15.54 ± 10.93	12.68 ± 7.81	−18.40	15	20 ± 14.06	17.58 ± 9.8	−12.10	**0.02**	0.07	0.84
HOMA-IR	48	1.86 ± 1.18	1.4 ± 0.88	−24.73	8	2.86 ± 1.73	2.53 ± 1.43	−11.54	0.06	**0.007**	0.74
QUICKI	43	0.34 ± 0.04	0.36 ± 0.04	5.88	7	0.32 ± 0.03	0.33 ± 0.03	3.13	**0.03**	0.07	0.70

TC: total cholesterol; HDL-C: high-density lipoprotein cholesterol; LDL-C: low-density lipoprotein cholesterol; TG: triglycerides; HOMA-IR: homeostatic model assessment for insulin resistance; QUICKI: quantitative insulin sensitivity check index; SD: standard deviation; %Δ: percentage variation; T × G: training × genotype interaction; the test applied was two-way mixed ANOVA.

**Table 2:** Models of multiple regression analysis before and after the physical exercise, with difference values in children and adolescents who are overweight/obese and normal weight.

Overweight and obese							
Dependent variable	Independent variables considered	Before (*n*=263)		After (*n*=135)		Difference (*n*=135)	
		*β *± SD	*p*	*β* ± SD	*p*	*β* ± SD	*p*
Glucose (mg/dl)	Genotype	0.06 ± 0.06	0.36	0.26 ± 0.09	**0.003**	−0.03 ± 0.09	0.76
	Age	0.07 ± 0.06	0.28	−0.03 ± 0.09	0.74	0.08 ± 0.09	0.35
	Sex	−0.06 ± 0.06	0.36	0.16 ± 0.09	0.07	−0.07 ± 0.09	0.42
	BMI *Z*-score (kg/m^2^)	0.08 ± 0.06	0.20	0.11 ± 0.09	0.23	−0.23 ± 0.09	**0.01**
	Type of physical exercise	—	—	−0.08 ± 0.09	0.38	−0.17 ± 0.09	0.07

Insulin (uIU/ml)	Genotype	−0.13 ± 0.06	**0.04**	−0.19 ± 0.09	**0.03**	−0.004 ± 0.10	0.97
	Age	0.16 ± 0.07	**0.01**	0.24 ± 0.09	**0.01**	−0.03 ± 0.10	0.79
	Sex	0.17 ± 0.06	**0.009**	0.11 ± 0.09	0.24	0.13 ± 0.10	0.20
	BMI *Z*-score (kg/m^2^)	0.35 ± 0.07	**10** ^–4^	0.36 ± 0.09	**0.0002**	−0.0008 ± 0.10	0.99
	Type of physical exercise	—	—	0.32 ± 0.09	**0.0005**	0.05 ± 0.10	0.60

HOMA-IR	Genotype	−0.24 ± 0.09	**0.01**	∗		∗	
	Age	0.14 ± 0.10	0.14				
	Sex	0.16 ± 0.09	0.09				
	BMI *Z*-score (kg/m^2^)	0.27 ± 0.10	**0.006**				
	Type of physical exercise	—	—				

QUICKI	Genotype	0.12 ± 0.09	0.20	∗		∗	
	Age	0.05 ± 0.1	0.59				
	Sex	−0.27 ± 0.09	**0.004**				
	BMI *Z*-score (kg/m^2^)	−0.38 ± 0.10	**0.0002**				
	Type of physical exercise	—	—				

Normal weight (*n*=169)							
Dependent variable	Independent variables considered	*β *± SD	*p*				

Glucose (mg/dl)	Genotype	−0.07 ± 0.08	0.37		
	Age	−0.04 ± 0.08	0.58		
	Sex	−0.24 ± 0.08	**0.002**		
	BMI *Z*-score (kg/m^2^)	0.02 ± 0.08	0.82		

Insulin (uIU/ml)	Genotype	−0.06 ± 0.07	0.39		
	Age	−0.03 ± 0.07	0.73		
	Sex	0.39 ± 0.07	**10** ^–4^		
	BMI *Z*-score (kg/m^2^)	0.10 ± 0.07	0.18		

HOMA-IR	Genotype	−0.12 ± 0.09	0.17		
	Age	0.17 ± 0.09	0.06		
	Sex	0.20 ± 0.09	**0.02**		
	BMI *Z*-score (kg/m^2^)	0.02 ± 0.09	0.79		

QUICKI	Genotype	0.09 ± 0.11	0.42		
	Age	0.19 ± 0.11	0.09		
	Sex	−0.14 ± 0.11	0.21		
	BMI *Z*-score (kg/m^2^)	−0.04 ± 0.11	0.70		

HOMA-IR: homeostatic model assessment for insulin resistance; QUICKI: quantitative insulin sensitivity check index; *β*: regression coefficient; SD: standard deviation; Genotype: TT + AT and AA (recessive model). ∗HOMA-IR and QUICKI after physical exercise and differences values have no sufficient variance for the regression analysis; therefore, no results are displayed.

## Supplementary Materials

Supplementary Materials Supplementary Table 1: biochemical variables means compared between carriers and noncarriers of AA genotype before and after the physical exercise in children and adolescents who are overweight and obese. Supplementary Table 2: biochemical variables means compared between carriers and noncarriers of AA genotype in children and adolescents who are normal weight.
