# Supplementary material for: FTO rs9939609 Does Not Interact with Physical Exercise but Influences Basal Insulin Metabolism in Brazilian Overweight and Obese Adolescents
Source: J Obes. 2018 Apr 26;2018:3134026. doi: 10.1155/2018/3134026 (PMC5944237; doi:10.1155/2018/3134026)
Supplement: Supplementary Materials — Supplementary Table 1: biochemical variables means compared between carriers and noncarriers of AA genotype before and after the physical exercise in children and adolescents who are overweight and obese. Supplementary Table 2: biochemical variables means compared between carriers and noncarriers of AA genotype in children and adolescents who are normal weight. [file 3134026.f1.docx]

**Supplementary Material**

**Supplementary Table 1**.Biochemical variables means compared between carriers and non-carriers of AA genotype before and after the physical exercise in overweight and obese children and adolescents.

| Overweightandobese | | | | | | | | | | | |
| --- | --- | --- | --- | --- | --- | --- | --- | --- | --- | --- | --- |
|  |  | Before | | |  |  |  | After | | | |
| Variables | N | TT+AT | N | AA | p |  | N | TT+AT | N | AA | p |
| TC (mg/dl) | 199 | 162.66 ± 35.86 | 30 | 165.35 ± 33.11 | 0.70 |  | 118 | 157.51 ± 32.92 | 17 | 148.3 ± 29.26 | 0.28 |
| HDL-C (mg/dl) | 224 | 47.26 ± 10.73 | 35 | 50.20 ± 11.14 | 0.10 |  | 117 | 46.46 ± 11.88 | 17 | 45.62 ± 9.91 | 0.89 |
| LDL-C (mg/dl) | 199 | 92.51 ± 29.50 | 30 | 90.17 ± 23.26 | 0.68 |  | 118 | 90.71 ± 27.41 | 17 | 82.62 ± 30.94 | 0.26 |
| TG (mg/dl) | 224 | 109.08 ± 57.87 | 35 | 104.87 ± 52.37 | 0.79 |  | 117 | 102.80 ± 50.29 | 17 | 98.06 ± 68.54 | 0.42 |
| Glucose (mg/dl) | 228 | 87.09 ± 10.12 | 35 | 85.37 ± 10.30 | 0.22 |  | 117 | 85.76 ± 8.06 | 18 | 79.57 ± 5.76 | **0.002** |
| Insulin (uUI/ml) | 191 | 13.82 ± 10.84 | 30 | 18.74 ± 16.25 | 0.07 |  | 94 | 12.68 ± 7.81 | 15 | 17.58 ± 9.80 | **0.04** |
| HOMA-IR | 88 | 1.64 ± 1.22 | 17 | 2.56 ± 1.59 | **0.006** |  | 48 | 1.40 ± 0.88 | 8 | 2.53 ± 1.43 | **0.01** |
| QUICKI | 81 | 0.36 ± 0.06 | 15 | 0.34 ± 0.08 | **0.04** |  | 43 | 0.36 ± 0.04 | 7 | 0.32 ± 0.03 | **0.04** |

TC: Total cholesterol; HDL-C: High-density lipoprotein cholesterol; LDL-C: Low-density lipoprotein cholesterol; TG: triglycerides; HOMA-IR: Homeostatic model assessment for insulin resistance; QUICKI: Quantitative insulin sensitivity check index; SD: Standard deviation; p: comparison between the initial and after 12 weeks means of physical exercise in overweight and obese children and adolescents.T test was applied to parametric variables and Mann-Whitney test was applied to no parametric variables.

**Supplementary Table 2**.Biochemical variables means compared between carriers and non-carriers of AA genotype in normal weight children and adolescents.

| Normal weight | | | | | |
| --- | --- | --- | --- | --- | --- |
| Variables | N | TT+AT | N | AA | p |
| TC (mg/dl) | 83 | 158 ± 29.46 | 5 | 154 ± 4.75 | 0.74 |
| HDL-C (mg/dl) | 142 | 47 ± 11.29 | 17 | 43 ± 9.43 | 0.21 |
| LDL-C (mg/dl) | 83 | 92 ± 26.95 | 5 | 92 ± 13.90 | 0.96 |
| TG (mg/dl) | 142 | 73.65 ± 31.44 | 17 | 78.74 ± 25.69 | 0.25 |
| Glucose (mg/dl) | 152 | 92 ± 11.96 | 17 | 94 ± 9.23 | 0.34 |
| Insulin (uUI/ml) | 142 | 5.57 ± 3.64 | 17 | 4.82 ± 5.92 | 0.92 |
| HOMA-IR | 110 | 1.12 ± 0.77 | 17 | 1.35 ± 1.09 | 0.64 |
| QUICKI | 73 | 0.41 ± 0.07 | 13 | 0.40 ± 0.09 | 0.29 |

TC: Total cholesterol; HDL-C: High-density lipoprotein cholesterol; LDL-C: Low-density lipoprotein cholesterol; TG: triglycerides; HOMA-IR: Homeostatic model assessment for insulin resistance; QUICKI: Quantitative insulin sensitivity check index; SD: Standard deviation; p: comparison between the initial and after 12 weeks means of physical exercise in overweight and obese children and adolescents. T test was applied to parametric variables and Mann-Whitney test was applied to no parametric variables.
